# An ETV6::NTRK3 fusion transcript in a core‐binding factor acute myeloid leukemia

**DOI:** 10.1002/jha2.706

**Published:** 2023-06-22

**Authors:** Lucie Coster, Françoise Huguet, Alban Canali, Jean‐Baptiste Rieu, Véronique Mansat‐ De Mas

**Affiliations:** ^1^ Haematology Laboratory Toulouse University Cancer Institute—Oncopole Toulouse France; ^2^ Haematology Department Toulouse University Cancer Institute—Oncopole Toulouse France; ^3^ Medical University Toulouse University III Jean‐Paul Sabatier Toulouse France

**Keywords:** acute myeloid leukemia, core‐binding factor leukemia, fusion transcript, translocation, tyrosine kinase inhibitor

1

An 88‐year‐old woman with no prior cancer history was referred to our center for rapidly evolving asthenia and dyspnea. Her full blood count revealed a dramatic hyperleukocytosis (white cell count 163 × 10^9^/L), associated with anemia (hemoglobin 104 g/L) and thrombocytopenia (platelet count 50 × 10^9^/L). Blood smear showed 89% monocytoid blastic cells (Figure 1). Flow cytometry study demonstrated a blast population positive for CD13, CD33, CD36, and CD64. Taken together, these features led to the conclusion that she was suffering from an acute monoblastic leukemia. Karyotyping revealed a three‐way translocation t(6;21;8)(q11;q22;q22), which was suspected to be a variant of the translocation t(8;21), one of the two drivers of core‐binding factor (CBF) leukemias. This translocation was associated with a trisomy 15 in our patient (Figure 1).

**FIGURE 1:**
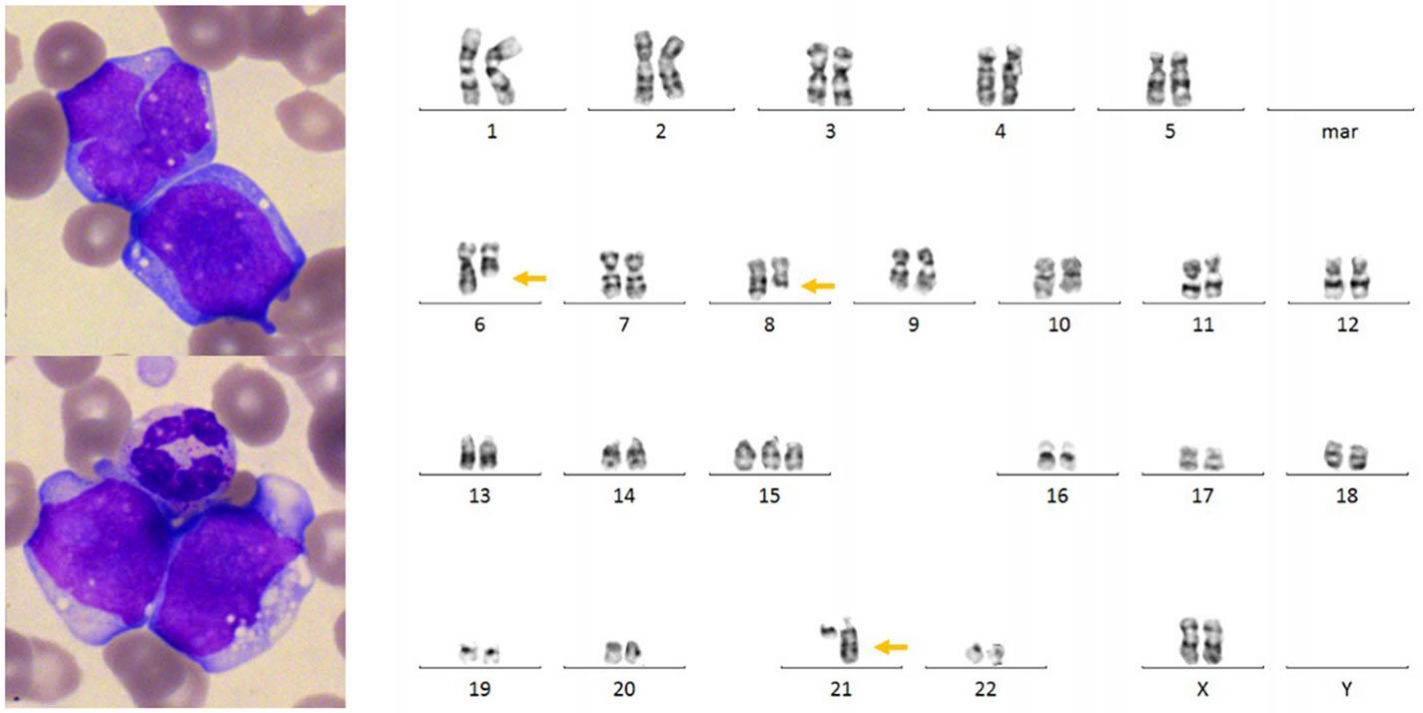
Blood smear showed blastic cells with irregular nuclei, dispersed chromatin and vacuolated cytoplasm suggestive of an acute myeloid leukemia of monocytic lineage (left panel, May Grünwald Giemsa stain, ×100 objective). A three‐way translocation t(6;21;8) was identified on the karyotype (yellow arrows) (right panel, G bands, Leishman stain, ×63 objective).

Reverse transcriptase multiplex ligation‐dependent probe amplification (RT‐MLPA) showed a RUNX1::RUNX1T1 fusion transcript, thus confirming the CBF leukemia and also revealed an ETV6::NTRK3 fusion transcript. Both those fusions were validated by fluorescence in situ hybridization (FISH) (Figure 2). Also, the derivative chromosome 15 of the translocation t(12;15) was demonstrated to be duplicated, as FISH showed that two acrocentric chromosomes bore a 3′ETV6 signal. After cytoreduction and two chemotherapy courses with azacitidine, palliative home care was set up at the patient's request.

**FIGURE 2:**
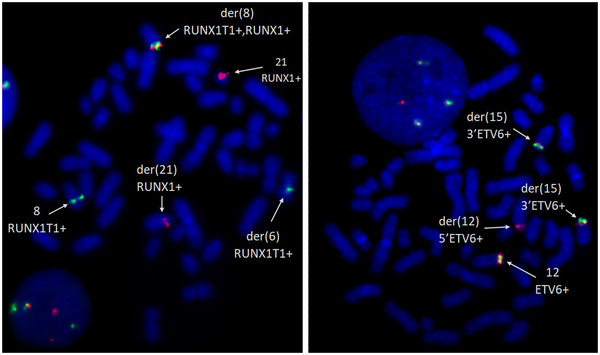
FISH showed a RUNX1::RUNX1T1 rearrangement (left panel, homemade RUNX1‐RUNX1T1 Texas Red—fluorescein probe, ×100 objective) and an ETV6 rearrangement (right panel, homemade ETV6 double color Texas Red—fluorescein probe, ×100 objective).

*ETV6* translocations in acute myeloid leukemias have been described as isolated or associated with a variety of translocations, notably translocations involving *NUP98* and *NUP214* genes. To our knowledge, this is the first description of an ETV6 translocation in a core‐binding factor acute myeloid leukemia (AML). Whether the presence of the ETV6::NTRK3 fusion gene worsens the good prognosis of t(8;21) AML is unknown. However, ETV6::NTRK3 is reported to be a target for tyrosine kinase inhibitors, which could restore a favorable prognosis.

## AUTHOR'S CONTRIBUTION

LC and VDM wrote the paper, FH provided the clinical data, JBR performed the flow cytometry analysis, AC executed the blood smear examination, LC conducted the cytogenetics study, and VDM carried out the molecular biology analysis.

## CONFLICT OF INTEREST STATEMENT

The authors declare that there is no conflict of interest.

### ETHICS APPROVAL STATEMENT

None.

## FUNDING SOURCES

None.

## Data Availability

None.

